# Supplementary material for: Understanding accelerators to improve SDG-related outcomes for adolescents—An investigation into the nature and quantum of additive effects of protective factors to guide policy making
Source: PLoS One. 2023 Jan 6;18(1):e0278020. doi: 10.1371/journal.pone.0278020 (PMC9821522; doi:10.1371/journal.pone.0278020)
Supplement: S1 Table — This table captures which accelerators an individual had access to. E.g., if a person is grouped under “CBO+FS”, this means they did not have access to CM, SC, and CP. (DOCX) [file pone.0278020.s001.docx]

**S1 Table**

| **Accelerator Combinations*** | **N (%)** |
| --- | --- |
| CBO Access (CBO) only | 10 (.6%) |
| Food Security (FS) only | 121 (7.0%) |
| Safe Community (SC) only | 65 (3.8%) |
| Caregiver Monitoring (CM) | 11 (.6%) |
| Caregiver Praise (CP) only | 106 (6.2%) |
| CBO+ FS | 45 (2.6%) |
| CBO+ SC | 7 (.4%) |
| CBO+ CM | 13 (.8%) |
| CBO+CP | 4 (.2%) |
| FS+SC | 139 (8.1%) |
| FS+CM | 21 (1.2%) |
| FS+CP | 138 (8.0%) |
| SC+CM | 27 (1.6%) |
| SC+CP | 82 (4.8%) |
| CM+CP | 12 (.7%) |
| CBO+FS+SC | 10 (.6%) |
| CBO+FS+CM | 71 (4.1%) |
| CBO+FS+CP | 29 (1.7%) |
| CBO+SC+CM | 7 (.4%) |
| CBO+SC+CP | 2 (.1%) |
| CBO+CM+CP | 7(.4%) |
| FS+SC+CM | 75 (4.4%) |
| FS+SC+CP | 208 (12.1%) |
| FS+CM+CP | 42 (2.4%) |
| SC+CM+CP | 12 (.7%) |
| CBO+FS+SC+CM | 39 (2.3%) |
| CBO+FS+SC+CP | 10 (.6%) |
| CBO+SC+CM+CP | 5 (.3%) |
| CBO+FS+CP+CM | 71 (4.1%) |
| FS+SC+CP+CM | 180 (10.5%) |
| CBO+FS+SC+CM+CP | 40 (2.3%) |
