# Supplementary material for: Understanding accelerators to improve SDG-related outcomes for adolescents—An investigation into the nature and quantum of additive effects of protective factors to guide policy making
Source: PLoS One. 2023 Jan 6;18(1):e0278020. doi: 10.1371/journal.pone.0278020 (PMC9821522; doi:10.1371/journal.pone.0278020)
Supplement: S2 Table — (DOCX) [file pone.0278020.s002.docx]

**S2 Table**

| **Outcomes/Provisions** | **Adjusted Probability (%)** | **Probability Difference to BL (95% CIs)** |
| --- | --- | --- |
| **No MDD (SDG 3.4)**  - No Accelerators | 65.50 |  |
| Food Security (FS) | 75.36 | + 9.86 [3.42; 16.29] |
| Safe Communities (SC) | 81.82 | +16.32 [10.47; 22.18] |
| Caregiver Praise (CP) | 75.84 | +10.34 [4.38; 16.30] |
| Caregiver Monitoring (CM) | 70.65 | + 5.15 [-2.21; 12.53] |
| CBO Access (CBO) | 84.90 | + 19.40 [11.18; 27.62] |
| FS + SC | 87.95 | + 22.45 [14.94; 29.96] |
| FS + CP | 83.54 | + 18.04 [10.04; 26.03] |
| FS + CM | 79.53 | + 14.03 [5.58; 22.48] |
| FS + CBO | 90.13 | + 24.63 [16.21; 33.05] |
| SC + CP | 88.23 | + 22.73 [15.46; 30.00] |
| SC + CM | 85.14 | + 19.64 [12.47; 26.80] |
| SC + CBO | 93.12 | + 27.62 [19.87; 35.38] |
| CP + CM | 79.95 | + 14.45 [6.25; 22.66] |
| CP + CBO | 90.37 | + 24.87 [16.48; 33.25] |
| CM + CBO | 87.75 | + 22.25 [14.19; 30.30] |
| FS + SC + CP | 92.42 | + 26.92 [18.89; 34.96] |
| FS + SC + CM | 90.29 | + 24.79 [16.87; 32.72] |
| FS + SC + CBO | 95.67 | + 30.17 [22.32; 38.03] |
| FS + CP + CM | 86.59 | + 21.09 [12.30; 29.89] |
| FS + CP + CBO | 93.86 | + 28.36 [19.97; 36.75] |
| FS + CM + CBO | 92.10 | + 26.60 [18.38; 34.82] |
| SC + CP + CM | 90.53 | + 25.03 [17.28; 32.78] |
| SC + CP + CBO | 95.78 | + 30.28 [22.51; 38.05] |
| SC+ CM + CBO | 94.53 | + 29.03 [21.44; 36.63] |
| CP + CM + CBO | 92.29 | + 26.79 [18.61; 34.96] |
| FS + SC + CP + CM | 93.97 | + 28.47 [20.36; 36.58] |
| FS + SC + CP + CBO | 97.38 | + 31.88 [24.12; 39.65] |
| FS + SC + CM + CBO | 96.58 | + 31.08 [23.36; 38.80] |
| FS + CP + CM + CBO | 95.13 | + 29.63 [21.45; 37.82] |
| SC +CP + CM + CBO | 96.67 | + 31.17 [23.53; 38.82] |
| All hypothesized accelerators | 97.94 | + 32.44 [24.80; 40.09] |
| **No Suicidal Ideation (SDG 3.4)** - No Accelerators | 89.04 |  |
| Food Security | 92.52 | + 3.48 [-1.04; 8.01] |
| Safe Communities | 92.53 | + 3.49 [-.93; 7.90] |
| Caregiver Praise | 95.28 | + 6.24 [2.12; 10.36] |
| Caregiver Monitoring | 91.35 | + 2.31 [-2.61; 7.22] |
| CBO Access | 94.90 | + 5.86 [.20; 11.52] |
| FS + SC | 94.98 | + 5.94 [.52; 11.34] |
| FS + CP | 96.87 | + 7.83 [2.73; 12.93] |
| FS + CM | 94.16 | + 5.12 [-.35; 10.59] |
| FS + CBO | 96.61 | + 7.57 [1.81; 13.33] |
| SC + CP | 96.87 | + 7.83 [2.82; 12.84] |
| SC + CM | 94.16 | + 5.12 [-.15; 10.40] |
| SC + CBO | 96.61 | + 7.57 [1.65; 13.50] |
| CP + CM | 96.34 | + 7.30 [2.48; 12.12] |
| CP + CBO | 97.91 | + 8.87 [3.65; 14.09] |
| CM + CBO | 96.05 | + 7.01 [1.52; 12.49] |
| FS + SC + CP | 97.94 | + 8.90 [3.49; 14.31] |
| FS + SC + CM | 96.11 | + 7.07 [1.43; 12.71] |
| FS + SC + CBO | 97.77 | + 8.73 [2.88; 14.57] |
| FS + CP + CM | 97.59 | + 8.55 [3.26; 13.83] |
| FS + CP + CBO | 98.63 | + 9.59 [4.22; 14.96] |
| FS + CM + CBO | 97.39 | + 8.35 [2.75; 13.94] |
| SC + CP + CM | 97.59 | + 8.55 [3.35; 13.74] |
| SC + CP + CBO | 98.63 | + 9.59 [4.22; 14.96] |
| SC+ CM + CBO | 97.39 | + 8.35 [2.69; 14.01] |
| CP + CM + CBO | 98.39 | + 9.35 [4.16; 14.55] |
| FS + SC + CP + CM | 98.42 | + 9.38 [3.96; 14.80] |
| FS + SC + CP + CBO | 99.11 | + 10.07 [4.67; 15.46] |
| FS + SC + CM + CBO | 98.28 | + 9.24 [3.59; 14.90] |
| FS + CP + CM + CBO | 98.95 | + 9.91 [4.60; 15.22] |
| SC +CP + CM + CBO | 98.95 | + 9.91 [4.61; 15.21] |
| All hypothesized accelerators | 99.32 | + 10.28 [4.96; 15.60] |
| **No PTSS (SDG 3.4)**  - No Accelerators | 91.45 |  |
| Food Security | 92.18 | + .73 [-2.76; 4.22] |
| Safe Communities | 94.18 | + 2.73 [-.23; 5.70] |
| Caregiver Praise | 95.75 | + 4.30 [1.51; 7.10] |
| Caregiver Monitoring | 85.22 | -6.23 [-11.88; -.58] |
| CBO Access | 91.77 | + .32 [-4.33; 4.98] |
| FS + SC | 94.69 | + 3.24 [-.85; 7.35] |
| FS + CP | 96.14 | + 4.69 [.89; 8.48] |
| FS + CM | 86.39 | - 5.06 [-11.33; 1.21] |
| FS + CBO | 92.48 | 1.03 [-4.36; 6.42] |
| SC + CP | 97.17 | + 5.72 [2.22; 9.21] |
| SC + CM | 89.67 | - 1.78 [-6.73; 3.18] |
| SC + CBO | 94.41 | + 2.96 [-2.09; 8.01] |
| CP + CM | 92.34 | + .89 [-3.42; 5.20] |
| CP + CBO | 95.93 | + 4.48 [.35; 8.60] |
| CM + CBO | 85.74 | - 5.71 [-12.92; 1.50] |
| FS + SC + CP | 97.43 | + 5.98 [1.92; 10.04] |
| FS + SC + CM | 90.54 | -.91 [-6.48; 4.66] |
| FS + SC + CBO | 94.91 | + 3.46 [-2.04; 8.95] |
| FS + CP + CM | 93.00 | + 1.55 [-3.50; 6.60] |
| FS + CP + CBO | 96.29 | + 4.84 [.17; 9.51] |
| FS + CM + CBO | 86.87 | - 4.58 [-11.83; 2.68] |
| SC + CP + CM | 94.81 | + 3.36 [-.90; 7.62] |
| SC + CP + CBO | 97.28 | + 5.83 [1.53; 10.14] |
| SC+ CM + CBO | 90.06 | -1.39 [-8.46; 5.70] |
| CP + CM + CBO | 92.64 | 1.19 [-4.40; 6.78] |
| FS + SC + CP + CM | 95.27 | + 3.82 [-1.02; 8.67] |
| FS + SC + CP + CBO | 97.53 | + 6.08 [1.44; 10.73] |
| FS + SC + CM + CBO | 90.90 | -.55 [-7.68; 6.58] |
| FS + CP + CM + CBO | 93.27 | 1.82 [-4.10; 7.75] |
| SC +CP + CM + CBO | 95.02 | + 3.57 [-1.85; 8.99] |
| All hypothesized accelerators | 95.46 | + 4.01 [-1.68; 9.71] |
| **Good Mental Health (SDG 3.4)** - No Accelerators | 59.07 |  |
| Food Security | 66.74 | + 7.67 [1.29; 14.07] |
| Safe Communities | 75.37 | + 16.30 [10.59; 22.01] |
| Caregiver Praise | 71.69 | + 12.62 [6.95; 18.29] |
| Caregiver Monitoring | 60.20 | + 1.13 [-5.83; 8.09] |
| CBO Access | 77.66 | + 18.59 [10.32; 26.87] |
| FS + SC | 81.01 | + 21.94 [14.23; 29.66] |
| FS + CP | 77.91 | + 18.84 [10.83; 26.86] |
| FS + CM | 67.78 | + 8.71 [.00; 17.43] |
| FS + CBO | 82.91 | + 23.84 [14.78; 32.89] |
| SC + CP | 84.36 | + 25.29 [18.10; 32.49] |
| SC + CM | 76.24 | + 17.17 [9.83; 24.51] |
| SC + CBO | 88.14 | + 29.07 [20.77; 37.37] |
| CP + CM | 72.64 | + 13.57 [5.51; 21.62] |
| CP + CBO | 85.99 | + 26.92 [18.37; 35.46] |
| CM + CBO | 78.47 | + 19.40 [10.82; 27.99] |
| FS + SC + CP | 88.29 | + 29.22 [20.91; 37.53] |
| FS + SC + CM | 81.73 | + 22.66 [14.17; 31.16] |
| FS + SC + CBO | 91.23 | + 32.16 [23.54; 40.79] |
| FS + CP + CM | 78.72 | + 19.65 [10.43; 28.86] |
| FS + CP + CBO | 89.56 | + 30.49 [21.53; 39.46] |
| FS + CM + CBO | 83.57 | + 24.50 [15.36; 33.64] |
| SC + CP + CM | 84.98 | + 25.91 [17.98; 33.85] |
| SC + CP + CBO | 92.96 | + 33.89 [25.76; 42.02] |
| SC+ CM + CBO | 88.63 | + 29.56 [21.26; 37.87] |
| CP + CM + CBO | 86.55 | + 27.48 [18.79; 36.17] |
| FS + SC + CP + CM | 88.78 | + 29.71 [21.04; 38.38] |
| FS + SC + CP + CBO | 94.88 | + 35.81 [27.50; 44.12] |
| FS + SC + CM + CBO | 91.61 | + 32.54 [23.93; 41.15] |
| FS + CP + CM + CBO | 90.00 | + 30.93 [21.92; 39.95] |
| SC +CP + CM + CBO | 93.27 | + 34.20 [26.04; 42.35] |
| All hypothesized accelerators | 95.11 | + 36.04 [27.72; 44.35] |
| **No Peer Problems (SDG 3.4)**  - No Accelerators | 10.39 |  |
| Food Security | 14.56 | + 4.17 [1.26; 7.07] |
| Safe Communities | 17.96 | + 7.57 [4.40; 10.75] |
| Caregiver Praise | 14.76 | + 4.37 [1.77; 6.97] |
| Caregiver Monitoring | 15.91 | + 5.52 [2.28; 8.76] |
| CBO Access | 33.33 | + 22.94 [16.02; 29.85] |
| FS + SC | 24.32 | + 13.93 [9.16; 18.70] |
| FS + CP | 20.27 | + 9.88 [5.63; 14.13] |
| FS + CM | 21.74 | + 11.35 [6.41; 16.28] |
| FS + CBO | 42.28 | + 31.89 [24.10; 39.66] |
| SC + CP | 24.62 | + 14.23 [9.33; 19.13] |
| SC + CM | 26.29 | + 15.90 [10.50; 21.30] |
| SC + CBO | 48.44 | + 38.05 [27.79; 48.32] |
| CP + CM | 22.02 | + 11.63 [6.58; 16.68] |
| CP + CBO | 42.67 | + 32.28 [23.24; 41.32] |
| CM + CBO | 44.83 | + 34.44 [26.45; 42.44] |
| FS + SC + CP | 32.38 | + 21.99 [16.06; 27.91] |
| FS + SC + CM | 34.33 | + 23.94 [17.65; 30.23] |
| FS + SC + CBO | 57.91 | + 47.52 [37.54; 57.51] |
| FS + CP + CM | 29.28 | + 18.89 [12.67; 25.10] |
| FS + CP + CBO | 52.15 | + 41.76 [32.66; 50.87] |
| FS + CM + CBO | 54.34 | + 43.95 [36.26; 51.64] |
| SC + CP + CM | 34.70 | + 24.31 [17.51; 31.12] |
| SC + CP + CBO | 58.31 | + 47.92 [36.83; 59.01] |
| SC+ CM + CBO | 60.43 | + 50.04 [40.49; 59.60] |
| CP + CM + CBO | 54.75 | + 44.36 [35.17; 53.55] |
| FS + SC + CP + CM | 43.77 | + 33.38 [26.46; 40.31] |
| FS + SC + CP + CBO | 67.20 | + 56.81 [46.73; 66.89] |
| FS + SC + CM + CBO | 69.11 | + 58.72 [50.32; 67.13] |
| FS + CP + CM + CBO | 63.92 | + 53.53 [45.30; 61.77] |
| SC +CP + CM + CBO | 69.46 | + 59.07 [49.59; 68.56] |
| All hypothesized accelerators | 76.93 | + 66.54 [58.47; 74.62] |
| **Prosocial Behaviour (SDG 3.4)** - No Accelerators | 29.85 |  |
| Food Security | 33.83 | + 3.98 [-.99; 8.94] |
| Safe Communities | 35.59 | + 5.74 [1.01; 10.48] |
| Caregiver Praise | 39.72 | + 9.87 [5.27; 14.47] |
| Caregiver Monitoring | 34.01 | + 4.16 [-.82; 9.14] |
| CBO Access | 61.31 | + 31.46 [23.98; 38.94] |
| FS + SC | 39.90 | + 10.05 [3.31; 16.78] |
| FS + CP | 44.18 | + 14.33 [7.55; 21.11] |
| FS + CM | 38.23 | + 8.38 [1.51; 15.26] |
| FS + CBO | 65.56 | + 35.71 [27.27; 44.15] |
| SC + CP | 46.11 | + 16.26 [9.49; 23.03] |
| SC + CM | 40.09 | + 10.24 [3.75; 16.74] |
| SC + CBO | 67.30 | + 37.45 [27.97; 46.93] |
| CP + CM | 44.38 | + 14.53 [7.59; 21.47] |
| CP + CBO | 71.05 | + 41.20 [32.77; 49.64] |
| CM + CBO | 65.75 | + 35.90 [28.36; 43.43] |
| FS + SC + CP | 50.72 | + 20.83 [12.73; 28.94] |
| FS + SC + CM | 44.56 | + 14.71 [6.99; 22.44] |
| FS + SC + CBO | 71.21 | + 41.36 [31.53; 51.18] |
| FS + CP + CM | 48.94 | + 19.09 [10.91; 27.27] |
| FS + CP + CBO | 74.68 | + 44.83 [35.88; 53.78] |
| FS + CM + CBO | 69.75 | + 39.90 [31.78; 48.02] |
| SC + CP + CM | 50.89 | + 21.04 [13.12; 28.95] |
| SC + CP + CBO | 76.13 | + 46.28 [36.75; 55.81] |
| SC+ CM + CBO | 71.37 | +41.52 [32.67; 50.38] |
| CP + CM + CBO | 74.83 | + 44.98 [36.87; 53.10] |
| FS + SC + CP + CM | 55.45 | + 25.60 [16.94; 34.26] |
| FS + SC + CP + CBO | 79.31 | + 49.46 [39.80; 59.11] |
| FS + SC + CM + CBO | 74.97 | + 45.12 [36.09; 54.16] |
| FS + CP + CM + CBO | 78.13 | + 48.28 [38.84; 56.73] |
| SC +CP + CM + CBO | 79.44 | +49.59 [40.79; 58.39] |
| All hypothesized accelerators | 82.28 | + 52.43 [43.55; 61.32] |
| **No Substance Abuse (SDG 3.5)** - No Accelerators | 92.86 |  |
| Food Security | 89.86 | - 3.00[-6.83; .82] |
| Safe Communities | 95.57 | + 2.71[.09; 5.31] |
| Caregiver Praise | 96.14 | +3.28 [.80; 5.75] |
| Caregiver Monitoring | 91.03 | -1.83 [-5.69; 2.03] |
| CBO Access | 95.70 | + 2.84 [-.58; 6.27] |
| FS + SC | 93.60 | + .74 [-3.40; 4.87] |
| FS + CP | 94.40 | +1.54 [-2.35; 5.43] |
| FS + CM | 87.39 | - 5.47 [-11.70; .74] |
| FS + CBO | 93.79 | + .93 [-4.16; 6.03] |
| SC + CP | 97.65 | + 4.79 [1.65; 7.92] |
| SC + CM | 94.37 | + 1.51 [-1.96; 4.98] |
| SC + CBO | 97.38 | + 4.52 [.87; 8.17] |
| CP + CM | 95.09 | +2.23 [-1.12; 5.58] |
| CP + CBO | 97.72 | + 4.86 [1.47; 8.26] |
| CM + CBO | 94.55 | + 1.69 [-2.36; 5.73] |
| FS + SC + CP | 96.55 | + 3.69 [-.33; 7.72] |
| FS + SC + CM | 91.93 | -.93 [-6.03; 4.17] |
| FS + SC + CBO | 96.16 | + 3.30 [-1.54; 8.15] |
| FS + CP + CM | 93.83 | + 0.97 [-4.92; 5.03] |
| FS + CP + CBO | 96.66 | + 3.80 [-.60; 8.21] |
| FS + CM + CBO | 92.18 | -.68 [-6.46; 5.09] |
| SC + CP + CM | 96.96 | +4.12 [.68; 7.58] |
| SC + CP + CBO | 98.62 | +5.76 [2.24; 9.29] |
| SC+ CM + CBO | 96.65 | + 3.79 [36.31; 49.39] |
| CP + CM + CBO | 97.08 | + 4.22 [.-.20; 7.77] |
| FS + SC + CP + CM | 95.61 | + 2.75 [-1.77; 7.27] |
| FS + SC + CP + CBO | 97.17 | + 5.11 [.90; 9.33] |
| FS + SC + CM + CBO | 95.12 | + 2.26 [-3.03; 7.56] |
| FS + CP + CM + CBO | 95.74 | + 2.88 [-1.95; 7.73] |
| SC +CP + CM + CBO | 98.23 | + 5.37 [1.67; 9.07] |
| All hypothesized accelerators | 97.40 | +4.54 [.14; 9.07] |
| **School attendance (SDG 4.1/4.4)** - No Accelerators | 97.57 |  |
| Food Security | 97.03 | -.54 [-2.47; 1.39] |
| Safe Communities | 97.70 | +.13 [-1.38; 1.64] |
| Caregiver Praise | 98.30 | +.73 [-.60; 2.06] |
| Caregiver Monitoring | 97.82 | + .25 [-1.21; 1.71] |
| CBO Access | 94.84 | -2.73 [-6.70; 1.23] |
| FS + SC | 97.19 | -.38 [-2.84; 2.08] |
| FS + CP | 97.91 | .34 [-1.79; 2.48] |
| FS + CM | 97.34 | -.23 [-2.66; 2.18] |
| FS + CBO | 93.74 | -3.83 [-8.74; 1.08] |
| SC + CP | 98.39 | .82 [-1.03; 2.68] |
| SC + CM | 97.94 | .37 [-1.50; 2.24] |
| SC + CBO | 95.11 | -2.46 [-7.58; 2.67] |
| CP + CM | 98.47 | .90 [-.77; 2.58] |
| CP + CBO | 96.35 | -1.22 [-4.82; 2.38] |
| CM + CBO | 95.36 | -2.21 [-5.92; 1.49] |
| FS + SC + CP | 98.02 | .45 [-2.06; 2.98] |
| FS + SC + CM | 97.48 | -.09 [-2.74; 2.56] |
| FS + SC + CBO | 94.06 | -3.51 [-9.65; 2.64] |
| FS + CP + CM | 98.13 | .56 [-1.78; 2.90] |
| FS + CP + CBO | 95.55 | -2.02 [-6.50; 2.46] |
| FS + CM + CBO | 94.35 | -3.22 [-7.65; 1.22] |
| SC + CP + CM | 98.56 | .99 [-1.00; 2.98] |
| SC + CP + CBO | 96.54 | -1.03 [-5.42; 3.36] |
| SC+ CM + CBO | 95.60 | -1.97 [-6.49; 2.55] |
| CP + CM + CBO | 96.72 | -.85 [-4.16; 2.46] |
| FS + SC + CP + CM | 98.23 | .66 [-1.91; 3.23] |
| FS + SC + CP + CBO | 95.79 | -1.78 [-7.09; 3.52] |
| FS + SC + CM + CBO | 94.65 | -2.92 [-8.22; 2.38] |
| FS + CP + CM + CBO | 96.00 | -1.57 [-5.60; 2.45] |
| SC +CP + CM + CBO | 96.00 | -.67 [-4.58; 3.23] |
| All hypothesized accelerators | 96.21 | -1.36 [-6.00; 3.28] |
| **In Right Grade for Age (SDG 4.4)** - No Accelerators | 54.34 |  |
| Food Security | 61.08 | + 6.74 [1.32; 12.17] |
| Safe Communities | 52.39 | -1.95 [-7.06; 3.16] |
| Caregiver Praise | 51.55 | -2.79 [-7.65; 2.07] |
| Caregiver Monitoring | 55.14 | + .80 [-4.58; 6.18] |
| CBO Access | 51.54 | -3.80 [-11.23; 3.64] |
| FS + SC | 59.20 | + 4.86 [-2.27; 12.00] |
| FS + CP | 58.39 | +4.05 [-3.04; 11.14] |
| FS + CM | 61.85 | + 7.51 [.38; 14.63] |
| FS + CBO | 57.40 | + 3.06 [-5.72; 11.84] |
| SC + CP | 49.60 | -4.74 [-11.72; 2.23] |
| SC + CM | 53.20 | -1.14 [-7.87; 5.59] |
| SC + CBO | 48.59 | -5.75 [-15.88; 4.37] |
| CP + CM | 52.36 | -1.98 [-9.02; 5.06] |
| CP + CBO | 47.75 | -6.59 [-15.94; 2.75] |
| CM + CBO | 51.36 | -2.98 [-10.74; 4.77] |
| FS + SC + CP | 56.47 | + 2.13 [-6.29; 10.59] |
| FS + SC + CM | 59.98 | + 5.64 [-2.32; 12.61] |
| FS + SC + CBO | 55.48 | +1.14 [-9.82; 12.10] |
| FS + CP + CM | 59.17 | +4.83 [-3.48; 13.14] |
| FS + CP + CBO | 54.65 | .31 [-10.06; 10.67] |
| FS + CM + CBO | 58.19 | 3.85 [-4.87; 12.57] |
| SC + CP + CM | 50.40 | -3.94 [-11.98; 4.10] |
| SC + CP + CBO | 45.80 | -8.54 [-20.05; 2.97] |
| SC+ CM + CBO | 49.39 | -4.95 [-14.82; 4.92] |
| CP + CM + CBO | 48.55 | -5.79 [-15.22; 3.65] |
| FS + SC + CP + CM | 57.27 | +2.93 [-6.05; 11.91] |
| FS + SC + CP + CBO | 52.70 | -1.64 [-13.88; 10.60] |
| FS + SC + CM + CBO | 56.28 | +1.94 [-8.51; 12.38] |
| FS + CP + CM + CBO | 55.45 | +1.11 [-9.04; 11.25] |
| SC +CP + CM + CBO | 46.60 | -7.74 [-18.90; 3.42] |
| All hypothesized accelerators | 53.50 | -.84 [-12.47; 10.79] |
| **Able to concentrate at school (SDG 4.1/4.4)** - No Acc. | 68.92 |  |
| Food Security | 81.78 | +12.86 [7.18; 18.54] |
| Safe Communities | 78.90 | + 9.98 [4.39; 15.57] |
| Caregiver Praise | 73.64 | + 4.72 [-.94; 10.39] |
| Caregiver Monitoring | 75.64 | + 6.72 [.39; 13.05] |
| CBO Access | 71.16 | + 2.24 [-7.04; 11.53] |
| FS + SC | 88.37 | + 19.45 [12.56; 26.43] |
| FS + CP | 85.00 | + 16.08 [8.87; 23.39] |
| FS + CM | 86.30 | + 17.38 [10.50; 24.27] |
| FS + CBO | 83.33 | +14.41 [5.81; 23.01] |
| SC + CP | 82.51 | + 13.59[6.29; 20.89] |
| SC + CM | 83.99 | +15.07 [8.30; 21.85] |
| SC + CBO | 80.63 | + 11.71 [1.62; 21.81] |
| CP + CM | 79.66 | + 10.74 [3.13; 18.35] |
| CP + CBO | 75.67 | +6.75 [-3.92; 17.41] |
| CM + CBO | 76.56 | + 7.64 [-.10; 17.38] |
| FS + SC + CP | 90.57 | + 21.65 [13.95; 29.35] |
| FS + SC + CM | 91.44 | + 22.52 [15.40; 29.65] |
| FS + SC + CBO | 89.44 | + 20.52 [11.77; 29.27] |
| FS + CP + CM | 88.84 | + 19.92 [12.26; 27.58] |
| FS + CP + CBO | 86.32 | + 17.40 [8.01; 27.79] |
| FS + CM + CBO | 87.53 | + 18.61 [10.58; 26.64] |
| SC + CP + CM | 86.88 | + 17.96 [10.27; 25.66] |
| SC + CP + CBO | 84.01 | + 15.09 [4.46; 25.72] |
| SC+ CM + CBO | 85.39 | + 16.47 [7.55; 25.39] |
| CP + CM + CBO | 81.34 | + 12.42 [2.70; 22.14] |
| FS + SC + CP + CM | 93.12 | + 24.20 [16.56; 31.83] |
| FS + SC + CP + CBO | 91.46 | + 22.54 [13.47; 31.61] |
| FS + SC + CM + CBO | 92.26 | + 23.34 [15.28; 31.39] |
| FS + CP + CM + CBO | 89.87 | + 20.95 [12.31; 29.59] |
| SC +CP + CM + CBO | 88.07 | + 19.15 [9.76; 28.54] |
| All hypothesized accelerators | 93.78 | + 24.86 [16.52; 33.02] |
| **No early sexual debut (SDG 5.6)** - No Accelerators | 90.46 |  |
| Food Security | 89.37 | -1.09 [-5.41; 3.22] |
| Safe Communities | 94.92 | + 4.46 [1.13; 7.79] |
| Caregiver Praise | 94.69 | + 4.23 [.97; 7.48] |
| Caregiver Monitoring | 95.65 | +5.19 [1.58; 8.79] |
| CBO Access | 93.70 | + 3.24 [-3.31; 9.81] |
| FS + SC | 94.38 | + 3.82 [-.82; 8.47] |
| FS + CP | 94.02 | + 3.56 [-1.12; 8.25] |
| FS + CM | 95.09 | + 4.63 [-.18; 9.44] |
| FS + CBO | 92.94 | + 2.48 [-5.34; 10.30] |
| SC + CP | 97.28 | + 6.82 [2.89; 10.74] |
| SC + CM | 97.79 | + 7.33 [3.58; 10.08] |
| SC + CBO | 96.75 | + 6.29 [1.16; 11.41] |
| CP + CM | 97.68 | + 7.22 [3.40; 11.04] |
| CP + CBO | 96.59 | + 6.13 [1.00; 11.26] |
| CM + CBO | 97.23 | + 6.77 [2.37; 11.17] |
| FS + SC + CP | 96.92 | + 6.46 [1.79; 11.13] |
| FS + SC + CM | 97.50 | + 7.04 [2.65; 11.43] |
| FS + SC + CBO | 96.32 | + 5.86 [-.12; 11.85] |
| FS + CP + CM | 97.37 | + 6.91 [2.41; 11.42] |
| FS + CP + CBO | 96.15 | + 5.69 [-.33; 11.71] |
| FS + CM + CBO | 96.86 | + 6.40 [1.22; 11.58] |
| SC + CP + CM | 98.85 | + 8.39 [4.45; 12.33] |
| SC + CP + CBO | 98.29 | + 7.83 [3.34; 12.32] |
| SC+ CM + CBO | 98.58 | + 8.12 [4.05; 12.26] |
| CP + CM + CBO | 98.55 | + 8.09 [3.95; 12.22] |
| FS + SC + CP + CM | 98.69 | + 8.23 [3.94; 12.53] |
| FS + SC + CP + CBO | 98.06 | + 7.60 [2.57; 12.63] |
| FS + SC + CM + CBO | 98.43 | + 7.97 [3.43; 12.51] |
| FS + CP + CM + CBO | 98.35 | + 7.89 [3.29; 12.49] |
| SC +CP + CM + CBO | 99.28 | + 8.82 [4.78; 12.87] |
| All hypothesized accelerators | 99.19 | +8.73 [4.43; 13.02] |
| **No violence perpetration (SDG 16.1)** - No Accelerators | 38.49 |  |
| Food Security | 39.12 | + .63 [-4.61; 5.87] |
| Safe Communities | 40.64 | + 2.15 [-2.70; 7.00] |
| Caregiver Praise | 47.20 | + 8.71 [4.01; 13.40] |
| Caregiver Monitoring | 44.53 | + 6.04 [.80; 11.28] |
| CBO Access | 47.24 | + 8.75 [1.50; 16.00] |
| FS + SC | 41.28 | + 2.79 [-4.11; 9.70] |
| FS + CP | 47.86 | + 9.37 [2.46; 16.28] |
| FS + CM | 45.19 | + 6.70 [-.37; 13.77] |
| FS + CBO | 47.90 | + 9.41 [.74; 18.08] |
| SC + CP | 49.43 | + 10.94 [4.21; 17.69] |
| SC + CM | 46.76 | + 8.27 [1.75; 14.80] |
| SC + CBO | 49.48 | + 10.99 [1.10; 20.88] |
| CP + CM | 53.42 | + 14.93 [8.08; 21.77] |
| CP + CBO | 56.11 | + 17.62 [8.61; 26.64] |
| CM + CBO | 53.46 | + 14.97 [7.41; 22.52] |
| FS + SC + CP | 50.10 | + 11.61 [3.42; 19.80] |
| FS + SC + CM | 47.42 | + 8.93 [1.12; 16.75] |
| FS + SC + CBO | 50.14 | + 11.65 [.87; 22.44] |
| FS + CP + CM | 54.07 | + 15.58 [7.44; 23.73] |
| FS + CP + CBO | 56.77 | + 18.28 [8.25; 28.30] |
| FS + CM + CBO | 54.12 | + 15.63 [7.06; 24.20] |
| SC + CP + CM | 55.64 | + 17.15 [9.41; 24.90] |
| SC + CP + CBO | 58.32 | + 19.83 [8.77; 30.88] |
| SC+ CM + CBO | 55.69 | + 17.20 [7.64; 26.75] |
| CP + CM + CBO | 62.14 | + 23.65 [14.80; 32.49] |
| FS + SC + CP + CM | 56.30 | + 17.81 [9.12; 26.49] |
| FS + SC + CP + CBO | 58.96 | + 20.47 [8.75; 32.18] |
| FS + SC + CM + CBO | 56.34 | + 17.85 [7.68; 28.02] |
| FS + CP + CM + CBO | 62.76 | + 24.27 [14.70; 33.84] |
| SC +CP + CM + CBO | 64.23 | + 25.74 [15.41; 36.08] |
| All hypothesized accelerators | 64.84 | + 26.35[15.55; 37.15] |
| **No caregiver abuse (SDG 16.2)** - No Accelerators | 36.74 |  |
| Food Security | 47.89 | + 11.15 [5.76; 16.53] |
| Safe Communities | 43.01 | + 6.27 [1.36; 11.17] |
| Caregiver Praise | 38.78 | + 2.04 [-2.56; 6.64] |
| Caregiver Monitoring | 43.57 | + 6.83 [1.49; 12.18] |
| CBO Access | 23.85 | -12.89 [-18.95; -6.84] |
| FS + SC | 54.42 | + 17.68 [10.65; 24.71] |
| FS + CP | 50.05 | + 13.31 [6.30; 20.32] |
| FS + CM | 54.99 | + 18.25 [11.02; 25.48] |
| FS + CBO | 33.18 | -3.56 [-11.80; 4.68] |
| SC + CP | 45.14 | + 8.40 [1.66; 15.14] |
| SC + CM | 50.08 | + 13.34 [6.68; 19.99] |
| SC + CBO | 28.95 | -7.79 [-16.55; .97] |
| CP + CM | 45.71 | 8.97 [2.05; 15.89] |
| CP + CBO | 25.47 | -11.27 [-19.03; -3.52] |
| CM + CBO | 29.43 | -7.31 [-14.13; -.49] |
| FS + SC + CP | 56.56 | + 19.82 [11.58; 28.06] |
| FS + SC + CM | 61.35 | + 24.61 [16.82; 32.40] |
| FS + SC + CBO | 39.22 | + 2.48 [-8.11; 13.07] |
| FS + CP + CM | 57.12 | + 20.38 [12.12; 28.64] |
| FS + CP + CBO | 35.13 | -1.61 [-11.34; 8.13] |
| FS + CM + CBO | 39.77 | + 3.03 [-5.48; 11.55] |
| SC + CP + CM | 52.24 | + 15.50 [7.61; 23.39] |
| SC + CP + CBO | 30.77 | -5.97 [-16.19; 4.25] |
| SC+ CM + CBO | 35.16 | -1.58 [-10.74; 7.58] |
| CP + CM + CBO | 31.27 | -5.47 [-13.90; 2.95] |
| FS + SC + CP + CM | 63.39 | +26.65 [18.00; 35.30] |
| FS + SC + CP + CBO | 41.31 | + 4.57 [-7.27; 16.40] |
| FS + SC + CM + CBO | 46.18 | +9.44 [-.88; 19.76] |
| FS + CP + CM + CBO | 41.87 | + 5.13 [-4.74; 14.99] |
| SC +CP + CM + CBO | 37.16 | +.42 [-10.09; 10.02] |
| All hypothesized accelerators | 48.33 | + 11.59 [.18; 23.01] |
