# Supplementary material for: Understanding accelerators to improve SDG-related outcomes for adolescents—An investigation into the nature and quantum of additive effects of protective factors to guide policy making
Source: PLoS One. 2023 Jan 6;18(1):e0278020. doi: 10.1371/journal.pone.0278020 (PMC9821522; doi:10.1371/journal.pone.0278020)
Supplement: Attachment — Submitted filename: Response to reviewers.docx [file pone.0278020.s004.docx]

Reviewer #2: Dear Authors,

Your responses address the revision comments. In addition, there are few more comments to the revised paper.

We would like to thank reviewer 2 for taking the time to re-review our manuscript and providing a valuable comment about generalizability, which we have now addressed in our manuscript as outlined below.

1. In the discussion section, you should discuss whether the results from the study in South Africa are generalizable to other regions/countries or are context specific for South Africa / Sub-Saharan Africa? If yes, to which other populations (geographically)? Is it so only for the synergistic interactions between accelerators or also for the precise factors studied and their distinct combinations? Especially, as the introduction starts with “Adolescents living in Sub-Saharan Africa…”, it is suggestive that the results are valid for this whole region even though the study subjects are only from South Africa (Do authors suggest that South Africa is representative for Sub-Saharan Africa?). In the first paragraph of discussion, though, LMIC are mentioned, leading to the conclusion that the results are valid for all LMIC countries….

We agree with the reviewer that caution against over-interpretation of our results is warranted. We have now made it clearer that the data are from South Africa and predominantly valid for this context. Given that there are both similarities and differences between South Africa and other Sub-Saharan African countries, we highlight that a replication in other countries is needed.

2. Keywords: Change “Policy” to “policy”.

In our version of the document, this is not capitalized. We are not sure why this was different in the document the reviewer received.

3. Figure 5: Label for y axis is missing (or title should be changed). What does 100 % in Figure 5 actually mean? Please keep in mind that some readers may first look at the Figure 5 and they should get enough comprehensive information from the figure alone on what it is about.

A label for the y axis has been added. Descriptive text has been added to the caption, clarifying the contents of the figure.

4. Supporting information: revise titles “S2 Appendix 2” and “S1 Table”, too add some order to the appendices.

In accordance with the labels recommended by the journal, we have named our Appendices S1 Table and S2 Table, which also reflects the table order. We had already updated this within the manuscript, but have now also labelled the supporting documents accordingly.

References

In accordance with the recommendations made by the editor, we have checked out reference list to ensure it is complete. We have updated two citations (15,28), which had previously been under review, with the publication details.
